# Etiogenic factors present in the cerebrospinal fluid from amyotrophic lateral sclerosis patients induce predominantly pro-inflammatory responses in microglia

**DOI:** 10.1186/s12974-017-1028-x

**Published:** 2017-12-16

**Authors:** Pooja-Shree Mishra, K. Vijayalakshmi, A. Nalini, T. N. Sathyaprabha, B. W. Kramer, Phalguni Anand Alladi, T. R. Raju

**Affiliations:** 1 Department of Neurophysiology, National Institute of Mental Health and Neurosciences (NIMHANS), Bangalore, 560029 India; 2 Present Address: Centre de Recherche CERVO, Quebec, QC G1J 2G3 Canada; 3 Department of Neurology, National Institute of Mental Health and Neurosciences (NIMHANS), Bangalore, 560029 India; 4 School of Mental Health and Neuroscience, Maastricht Universitair Medisch Centrum, Maastricht, Limburg Netherlands

**Keywords:** Sporadic ALS, Microglia, ALS-CSF, Neuroinflammation, Non-cell autonomous pathology, Chitotriosidase, IL-6, IL-10, TNF-α, IFN-γ, PGE2, COX-2, iNOS, Arginase, VEGF, GDNF

## Abstract

**Background:**

Microglial cell-associated neuroinflammation is considered as a potential contributor to the pathophysiology of sporadic amyotrophic lateral sclerosis. However, the specific role of microglia in the disease pathogenesis remains to be elucidated.

**Methods:**

We studied the activation profiles of the microglial cultures exposed to the cerebrospinal fluid from these patients which recapitulates the neurodegeneration seen in sporadic amyotrophic lateral sclerosis. This was done by investigating the morphological and functional changes including the expression levels of prostaglandin E2 (PGE2), cyclooxygenase-2 (COX-2), TNF-α, IL-6, IFN-γ, IL-10, inducible nitric oxide synthase (iNOS), arginase, and trophic factors. We also studied the effect of chitotriosidase, the inflammatory protein found upregulated in the cerebrospinal fluid from amyotrophic lateral sclerosis patients, on these cultures.

**Results:**

We report that the cerebrospinal fluid from amyotrophic lateral sclerosis patients could induce an early and potent response in the form of microglial activation, skewed primarily towards a pro-inflammatory profile. It was seen in the form of upregulation of the pro-inflammatory cytokines and factors including IL-6, TNF-α, iNOS, COX-2, and PGE2. Concomitantly, a downregulation of beneficial trophic factors and anti-inflammatory markers including VEGF, glial cell line-derived neurotrophic factor, and IFN-γ was seen. In addition, chitotriosidase-1 appeared to act specifically via the microglial cells.

**Conclusion:**

Our findings demonstrate that the cerebrospinal fluid from amyotrophic lateral sclerosis patients holds enough cues to induce microglial inflammatory processes as an early event, which may contribute to the neurodegeneration seen in the sporadic amyotrophic lateral sclerosis. These findings highlight the dynamic role of microglial cells in the pathogenesis of the disease, thus suggesting the need for a multidimensional and temporally guarded therapeutic approach targeting the inflammatory pathways for its treatment.

**Electronic supplementary material:**

The online version of this article (10.1186/s12974-017-1028-x) contains supplementary material, which is available to authorized users.

## Background

The exact pathomechanism of neurodegeneration in amyotrophic lateral sclerosis (ALS), esp. the sporadic forms of ALS, remains poorly understood, thus leading to the lack of an effective therapeutic intervention for the disease [1, 2]. Several studies have reported a non-cell autonomous glial involvement in the pathogenesis, and the relevance of innate as well as adaptive immunity in ALS has also been widely discussed [3]. However, the requirement of the dynamic modulatory signals from within the central nervous system (CNS) for the recruitment and regulation of the adaptive immunity across the blood-brain barrier (BBB)/brain-spinal cord barrier (BSCB) further highlights the importance of the resident immune cells of the CNS in the disease pathology [4]. Microglia, the specialized immune cells of the CNS, constantly survey and dynamically regulate the neuronal milieu in the healthy and diseased CNS [5]. Depending on the nature and extent of the insult, microglial cells have been proposed to adopt morphologically and functionally distinct reactive phenotypes. These distinct phenotypes may perform diversified functions ranging from facilitating the pro-inflammatory processes that promote neuroinflammation to inducing an anti-inflammatory process that is engaged in healing and wound repair [6]. A fine balance among these phenotypes is considered to be crucial for a competent surveillance system, and its disruption could lead to the self-propagating chronic neuroinflammation seen in several neurological disorders [7]. However, the typical classification based on microglial polarization into classically (M1) and alternatively (M2) activated microglia is controversial and has been parallelly challenged [8].

Activated microglia have previously been reported in the autopsy samples and animal models of familial ALS (FALS), as well as throughout the symptomatic stages as demonstrated by neuroimaging of the ALS patients [9–11]. While many studies report microglial activation to be actively neurotoxic in ALS [12–14], there are also studies that report the microglial involvement to ne either neuroprotective or having no significant role to play in the event of neurodegeneration in such ALS models [15, 16]. Further, the spatial microglia appeared to affect their activation status in these animal models [17]. Incidentally, majority of these studies were conducted with the transgenic models containing superoxide dismutase 1 (SOD1) mutations that are uncommon in sporadic ALS (SALS) (> 1%) [18]. In recent years, the focus has largely shifted to transgenic models with novel, gene mutations more commonly reported in ALS patients, thus narrowing the gap between animal models and actual disease etiopathogenesis. Of prominence are ubiquitinated cytoplasmic inclusions (~ 98%)/mutations (FALS 5%, SALS 1%) in 43-kDa TAR DNA-binding protein (TDP-43), fused in sarcoma (FUS) (FALS 4%, SALS < 1%) and, most prominently, the hexaneucleotide repeat expansion in C9orf72 (FALS 40%, SALS > 10%) [19–21]. However, while these models recapitulate the etiopathogenesis of FALS more effectively, their relevance from the perspective of SALS pathogenesis, which constitutes 90% of the ALS etiology, is still dubious [22]. Although the emergence of these models has opened newer research avenues including aberrant RNA processing and protein degradation pathways, as well as perturbed nucleocytoplasmic transport [21], the neuroinflammatory pathways, esp. in SALS remain unraveled. Moreover, it remains unclear whether the microglial cell-mediated neurotoxicity or protection targeted the mutated protein or mimicked the overall disease pathology. The recent in vitro approaches targeting the induced pluripotent stem cells from SALS patients have been a significant step, but majority of the studies conducted till date have focused on the motor neuron pathology, precluding their relevance to microglial contribution [23, 24].

Therefore, we studied the effect of the disease-related factors circulated in the cerebrospinal fluid of ALS patients (ALS-CSF) on microglia to understand the direct nature of the insult in the SALS pathogenesis. Our hypothesis was based on the assumption that ALS-CSF may provide a mirror to the molecular effectors being produced and propagated in the diseased CNS, which may lead to progression of the disease. The neurodegenerative potential of ALS-CSF has already been documented in a plethora of molecular, electrophysiological, and behavioral studies, and the efficacy of the model in recapitulating the ALS pathology has been suggested [25–32]. Our previous investigations that aimed at determining the putative toxic agent(s) by the proteomic analysis of ALS-CSF demonstrated multifold upregulation of chitotriosidase-1 (CHIT-1), an inflammatory protein secreted by activated macrophages [33, 34]. Therefore, we investigated the response of microglia towards the ALS-CSF, as well as towards CHIT-1 in doses comparable to those found in the CSF samples taken for the study [33]. Microglial cultures exposed to ALS-CSF were analyzed for the production of toxic factors including free radicals and nitric oxide release as well as for inflammatory cytokines interleukin-6 (IL-6), interleukin-10 (IL-10), interferon-γ (IFN-γ), and tumor necrosis factor-α (TNF-α), Cyclooxygenase-2 (COX-2), and prostaglandin E2 (PGE2). In order to determine the trophic modulation in microglia in response to ALS-CSF, the changes in the expression patterns of vascular endothelial growth factor (VEGF) and glial cell line-derived neurotrophic factor (GDNF) were studied.

## Methods

### Diagnosis, CSF collection, and exposure

Diagnosis of ALS was based on the revised El Escorial criteria [35]. CSF samples from five drug-naive SALS patients (ALS-CSF) (Table 1, Additional file 1) and the age-matched disease control (patients suffering from neurological diseases, except neuroinflammatory and neurodegenerative diseases; NALS-CSF) (Table 2, Additional file 1) were collected after obtaining informed consent in accordance with the institutional human ethics committee guidelines. For ALS-CSF, careful screening of the family history of the patients was done to rule out FALS. The mean duration of illness taken into the consideration for the study was 12 ± 6 months, with mild (40%) to moderate disease severity (60%) and limb (40%), as well as bulbar (60%) onset patterns. The samples thus obtained were snap frozen in liquid nitrogen and stored at − 80 °C until further use.

**Table 1:** List of the antibodies used in the study

Antibody	Manufacturer	Ratio	Time (h)	Temperature (°C)
Primary antibody				
Anti-arginase rabbit polyclonal	Abcam	1:500	24	4
Anti-CHIT-1 rabbit polyclonal	Abcam	1:500	24	4
Anti-COX-2 rabbit polyclonal	Abcam	1:800	24	4
Anti-GDNF mouse monoclonal	SCBT	1:200	24	4
Anti-Iba-1 goat polyclonal	Abcam	1:800	24	4
Anti-IFN-γ goat polyclonal	SCBT	1:200	24	4
Anti-IL-10 rabbit polyclonal	Abcam	1:500	24	4
Anti-IL-6 rabbit polyclonal	Abcam	1:500	24	4
Anti-iNOS rabbit polyclonal	Abcam	1:800	24	4
Anti-PGE2 rabbit polyclonal	Abcam	1:500	24	4
Anti-TNF-α mouse monoclonal	Abcam	1:200	24	4
Anti-VEGF rabbit polyclonal	Abcam	1:500	24	4
Secondary antibody				
Anti-rabbit IgG (FITC-conjugated)	Chemicon	1:200	2	RT
Anti-rabbit IgG (FITC-conjugated)	Chemicon	1:200	2	RT
Anti-rabbit IgG (Cy3-conjugated)	Sigma-Aldrich	1:200	2	RT
Anti-mouse IgG (Cy3-conjugated)	Sigma-Aldrich	1:200	2	RT
Anti-goat IgG (Cy3-conjugated)	Sigma-Aldrich	1:200	2	RT
Anti-goat IgG (Cy3-conjugated)	Sigma-Aldrich	1:200	2	RT
Anti-rabbit IgG (Cy3-conjugated)	Sigma-Aldrich	1:200	2	RT
Anti-rabbit IgG (FITC-conjugated)	Chemicon	1:200	2	RT
Anti-rabbit IgG (Cy3-conjugated)	Chemicon	1:200	2	RT
Anti-rabbit IgG (FITC-conjugated)	Chemicon	1:200	2	RT
Anti-mouse IgG (Cy3-conjugated)	Sigma-Aldrich	1:200	2	RT
Anti-rabbit IgG (FITC-conjugated)	Chemicon	1:200	2	RT

**Table 2:** List of the primers and hydrolysis probes used in the study

Gene	Sense primer	Anti-sense primer	Probe
Arginase	AGCTGAGCAGCTGGACAG	CTCCGATAATCTCTATGGGCTTTGG	AGCAGCAGCAGCAGCAGGAACC
COX-2	CCAACCTCTCCTACTACACCAG	GTTCCTTATTTCCTTTCACACCCATG	CCTTCCTCCTGTGGCTGATGACTGC
GDNF	CGCCGGTAAGAGGCTTCTC	GATAATCTTCGGGCATATTGGAGTC	CGCCCGCCGAAGACCACTCCCT
IFN-γ	GCACAAAGCTGTCAATGAACTCA	CCAGAATCAGCACCGACTCC	CTGTCACCAGAATCTAGCCTAAGGAAGCGG
IL-10	CATGGCCTTGTAGACACCTTTG	CATCGATTTCTCCCCTGTGAGA	TCATTCTTCACCTGCTCCACTGCCTTGCTT
IL-6	TCCAGCCAGTTGCCTTCTTG	TCCTCTGTGAAGTCTCCTCTCC	ACTGATGTTGTTGACAGCCACTGCCTTCC
iNOS	CATCGACCTGGGCTGGAA	CCTCTGGATCTTGACCGTGAG	CGATGTGCTGCCTCTGGTCCTGC
TNF-α	TGGCGTGTTCATCCGTTCTCTA	CTCTGAGGAGTAGACGATA	TGGCGTGTTCATCCGTTCTCTA
VEGF	GAGCAACGTCACTATCGAGATC	GGCTTTGTTCTATCTTTCTTGGTC	TGCCGATCAAACCTCACCAAAGCCA

#### Study groups

For the experiments involving ALS-CSF exposure, the cultures were supplemented with 10% *v*/*v* CSF in Dulbecco’s modified Eagle’s medium (DMEM) and the effects were studied in duplicates or triplicates for individual samples. The study consisted of the following experimental groups:

- Normal control (NC): cultures propagated in DMEM alone
- NALS (disease control): cultures exposed to CSF of the NALS (10% *v*/*v* in DMEM)
- ALS: cultures exposed to ALS-CSF (10% *v*/*v* in DMEM)

For the experiments related to studying the effect of microglial cell-conditioned media (MCM), the groups were as follows:

- NC-MCM: NSC-34 cultures grown in the conditioned media from normal control microglial cultures
- NALS-MCM: NSC-34 cultures grown in the conditioned media from NALS group
- ALS-MCM: NSC-34 cultures grown in the conditioned media from the ALS group

For the study concerning exposure to CHIT-1, the experimental groups were as follows:

- NC: cultures propagated in DMEM
- Buffer control (buffer): cultures exposed to buffer (10% *v*/*v* of DMEM)
- CHIT-1: cultures exposed to CHIT-1 in a dose equivalent to that found in ALS-CSF [33] (18 pg/μl; 10% *v*/*v*, DMEM)

### Cell culture

#### Mixed glial cultures and enriched astroglial cultures

Spinal cords of Wistar rat pups (P2–P3) were dissected, freed of meninges, and mechanically triturated in DMEM. The single cell suspension thus obtained was propagated in DMEM with 10% FBS (Gibco BRL), with a seeding density of 2.5 × 10^4^ cells/ml. The enriched primary astroglial cultures were obtained from the mixed glial cultures using a previously described protocol [36]. Both the mixed glial and enriched astroglial cultures were allowed to attain a confluence of 70–80%, following which the exposure to ALS-CSF or CHIT-1, along with appropriate control groups, was carried out.

#### Microglial cultures

Enriched microglial cultures were obtained using a method by Saura and colleagues to ensure the purity and yield [37]. Briefly, upon reaching confluence (15–20 DIV), the mixed glial cultures were subjected to mild trypsin treatment (trypsin/EDTA DMEM, 1:4) to remove the astroglial monolayer, leaving intact the microglial layer growing beneath the astroglial monolayer. The purity of the microglial cultures was determined to be ≥ 99% using the microglial marker Iba-1, nuclear marker TO-PRO-3, and astroglial marker glial fibrillary acidic protein (GFAP). The enriched cultures were then subjected to the experimental treatments within 24 h.

#### NSC-34 cultures

The NSC-34 motor neuronal cell line was procured (Cedarlane, Canada) and maintained in accordance with the published protocol [27]. To study the effect of conditioned media, NSC-34 cells were plated at a density of 2.5 × 10^4^ cells/ml and allowed to reach 70–80% confluence. The cultures were then subjected to different experimental conditions.

### Immunofluorescence

Expression and localization of CHIT-1 were determined by qualitative observations on immunofluorescently labeled cells. A qualitative, as well as quantitative, approach was taken for the localization and analysis of cytokines, inflammatory markers, and trophic factors [36]. Briefly, mixed glial and enriched microglial cultures were plated onto poly-l-lysine-coated 13-mm circular coverslips (0.1 mg/ml). The cultures were then exposed to the ALS-CSF or CHIT-1 experimental groups and fixed with 4% paraformaldehyde (PFA) for 15 min at RT. Following blocking with 3% bovine serum albumin (BSA), the cultures were subsequently incubated with primary antibodies of interest followed by fluorescently labeled appropriate secondary antibodies (FITC or Cy3) (Table 1). TO-PRO-3-iodide (TOPRO) was used to stain the nucleus. The coverslips were then mounted using PVA-DABCO (Sigma-Aldrich, USA) and viewed under the laser scanning confocal microscope (Leica TCS SL, Germany), with excitation wavelengths at 488, 514, and 633 nm for FITC, Cy3, and TOPRO, respectively. In the case of double immunofluorescence, the antibodies raised in different animals were chosen and emission frequencies were segregated to avoid non-specific overlap of labeling.

#### Measurement of protein expression

We quantified the cellular expression of proteins of interest in terms of mean fluorescence intensity of individual cells on the images captured by a confocal microscope for each immunolabeled protein, using the inbuilt software of Leica Microsystems [36, 38, 39]. Briefly, 8-bit images were captured at × 20 magnifications with a constant PMT voltage, from randomly selected ten non-overlapping fields on each coverslip. Other parameters like optical zoom, frame and line average, resolution, frame (1024 × 1024), and exposure time were kept constant. The fluorescence intensity was measured on a scale of 0–255, and the immunostained region of each cell (region of interest, ROI) was demarcated using the *polyline* profile of the software. The area from 20 such cells or ROI, thus randomly marked, was quantified for each image, following which the fluorescence intensities were generated as numerical values commensurate to the staining. Ten such images were quantified for a single replicate of a sample. Five such samples were analyzed in duplicates for each group. Background reduction was applied for each analysis, and the mean fluorescence intensities (MFIs) were then compared between the study groups.

#### Measurement of soma area

For the measurement of soma area, the contour of cells was carefully drawn using the polyline profile in the same manner as described above. The report was generated for the soma area for each ROI, and the mean area per image was analyzed and compared within the groups.

#### Morphological transformation studies

After 48 h of ALS-CSF exposure, microglial cultures were observed and quantified for the morphological diversity ranging from ramified/resting to amoeboid/activated cells in randomly chosen fields, by phase contrast, as well as by Iba-1 immunofluorescence. Ten such fields were analyzed in duplicates for each study group, and the results were compared.

### Estimation of secreted cytokines

For a temporal analysis, the cultures were exposed to CSF for 12, 24, and 48 h, respectively, and the decanted media from the cultures were used for the estimation. Quantitative analysis of the secreted IL-6, IL-10, IFN-γ, and TNF-α levels was performed using specific rat ELISA kits and according to the manufacturer’s instructions (Ray Biotech, Inc., Georgia, USA).

### Glutamate estimation

For glutamate estimation, the medium from the normal controls and each of the subsets exposed to ALS-CSF for 12, 24, and 48 h was collected and centrifuged at 14,000 rpm to remove the cellular debris. Glutamate levels were measured using the glutamate assay kit (MAK004; Sigma-Aldrich, USA). The measurement was based on enzymatic conversion, and the absorbance was read at 450 nm using a colorimetric ELISA microplate plate reader (Tecan 2500 fluorometer, USA).

### ROS measurement

After CSF exposure for 48 h, the cultures were treated with dichlorofluorescin diacetate (DCFDA), harvested, and lysed and the resultant fluorescence release was measured at the excitation/emission spectrum of 480/530 nm in the 96-well plate reader (Tecan 2500 fluorometer, USA). Fluorescence/cell was calculated (arbitrary fluorescence unit, AFU) and statistically analyzed. Another set of cultures grown on 13-mm coverslips was incubated with DCFDA at 37 °C for 30 min and directly viewed (excitation = 480 nm; emission = 530 nm) under a confocal laser microscope.

### Nitric oxide measurement

Media from different experimental groups were collected as previously discussed. In addition, lysates were prepared from the microglial cultures after 48 h of exposure to ALS-CSF. The level of nitrates was assayed in the samples using a nitric oxide assay kit (AB65328; Abcam) at 540 nm using the colorimetric ELISA microplate reader.

### Cell viability and toxicity assay

Microglial cultures and NSC-34 cells seeded on 96-well plates (500 cells/well) were subjected to different experimental conditions, and MTT (3-(4,5-dimethylthiazol-2-yl)-2,5-diphenyltetrazolium bromide) and/or lactate dehydrogenase (LDH) assays were performed on these cultures and/or culture media according to the previously published protocols [27].

### Quantitative RT-PCR

Following exposure to CSF for 48 h, total RNA was extracted from the microglial cultures using the RNeasy Plus Mini Kit (Qiagen, USA) and quantified using 1 μl of RNA in a NanoDrop 2000 spectrophotometer (Thermo Fisher Scientific, Inc.). Total RNA (10 ng) was reverse transcribed (RT) with random primers using high-capacity cDNA reverse transcription kit (Applied Biosystems, USA). Amplification was carried out in triplicates using specific primers and hydrolysis probes for the target genes (Eurogentec, Belgium, Table 2), and reaction efficiencies for the target genes were analyzed and compared to 18S messenger RNA (mRNA) (endogenous control) using the standard curve method, so that a PCR efficiency of ~ 100 ± 10% was achieved. The cycle threshold (Ct) values were normalized to the endogenous control 18S mRNA, and the relative fold change was calculated using the comparative CT method (ΔΔCT method) [40].

### Statistical analysis

The data on mRNA expression and quantitative immunofluorescence was statistically assessed for significance by one-way ANOVA followed by Tukey’s post hoc test. For ELISA, glutamate, NO, ROS, and MTT assays, the statistical analysis was carried out using Student’s *t* test. GraphPad Prism was the software used to determine the statistical significance and *P* values for each experiment.

## Results

### ALS-CSF activated microglia, and the response was dynamic as well as temporally regulated

**Fig. 1:**
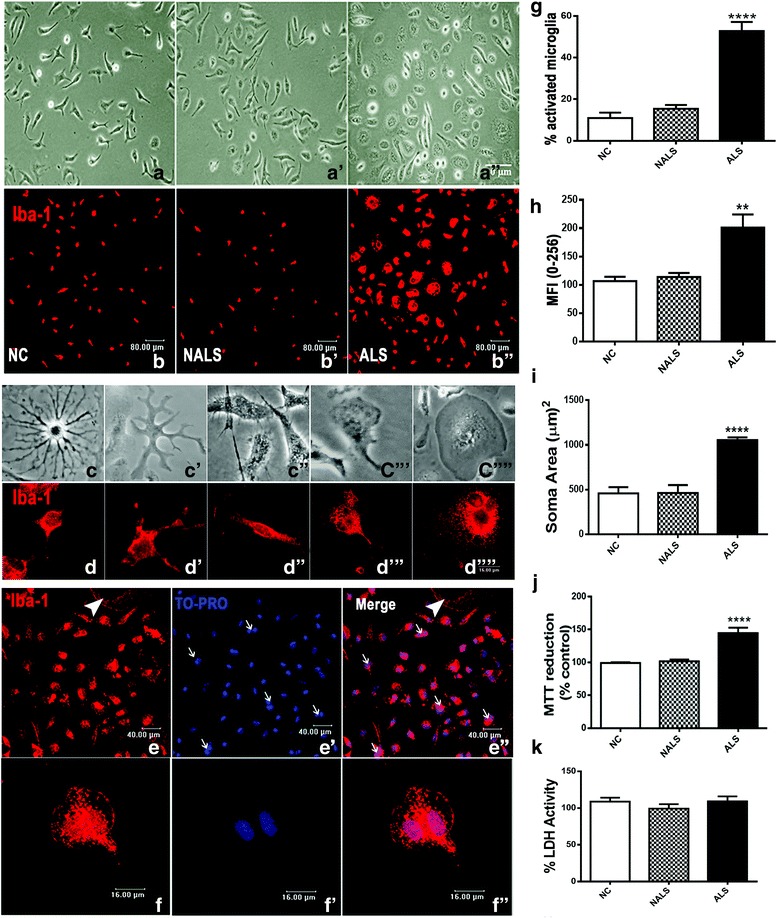
Microglial cells are activated in response to ALS-CSFCSF: When compared to NC (**c**,**d**) and NALS (**a’**, **b’**), note the increased number of amoeboid microglia upon exposure to ALS-CSF (**a’’**, **b’’**). Representative phase contrast (**c**–**c””**) and confocal images immunolabeled for Iba-1 (**d**–**d””**) of the different morphologies of the microglial cells in cultures. **c** and **d** represent the ramified microglial cells, while **c’**–**c”’** and **d’**–**d”’** the intermediate stages between ramified and amoeboid morphology. **c””** and **d’”** represent the fully activated amoeboid micro glial cells. The number of amoeboid microglial cells (**g**, ****p <0.0001 NC and NALS vs. ALS; *n*=6 in triplicates), the soma area (**h**, ****p <0.0001 NC vs. ALS; n=5 in duplicates) and Iba-1 expression (**i**, **p <0.01 NC and NALS vs. ALS; *n*=5 in duplicates) was significantly higher in the ALS group as compared to the NALS and control groups. The microglial cells exposed to ALS-CSF showed an increase in the no. of viable cells as seen by the MTT reduction assay, while no significant change was observed in the secreted LDH levels (**j** and **k**, respectively). Apart from the amoeboid morphology, many cells also showed multinucleation (**e’**, **e’’** white arrow). Nuclear labelling was carried out with TO-PRO (Blue, **e’** **f’**). The arrows represent the nuclei present in a single microglial cell. Also, cells with the disintegrating cell membrane (cytorrhexis) were fairly evident in random fields at 48h of ALS-CSF exposure (**e**-**f’’** white arrowheads). Scale bars are indicated. MFI=mean fluorescence intensity

Upon exposure to ALS-CSF, the microglial cells showed dynamic structural responses, adopting various morphologies ranging from the ramified to amoeboid morphology (Fig. 1a–d”). The increasing number of cells showing retraction of processes with a highly granular and distinctively enlarged soma suggested the adoption of a motile phagocytic phenotype in response to the etiogenic factors present in ALS-CSF (Fig. 1g, i). The response was specific to ALS-CSF, as the disease controls (NALS) did not induce this phenomenon. Apart from the amoeboid morphology, we also found multinucleated giant microglial cells (MGCs) predominantly in the cultures exposed to ALS-CSF (Fig. 1e–f”). Some cells also showed a disintegrated cytoplasm, resembling the process of cytorrhexis [41] (Fig. 1e–e”, white arrowheads). Increased Iba-1 expression and enhanced MTT reduction in cultures exposed to ALS-CSF for 48 h indicated sustained activation, possibly leading to microglial proliferation (Fig. 1h, j, k).

**Fig. 2:**
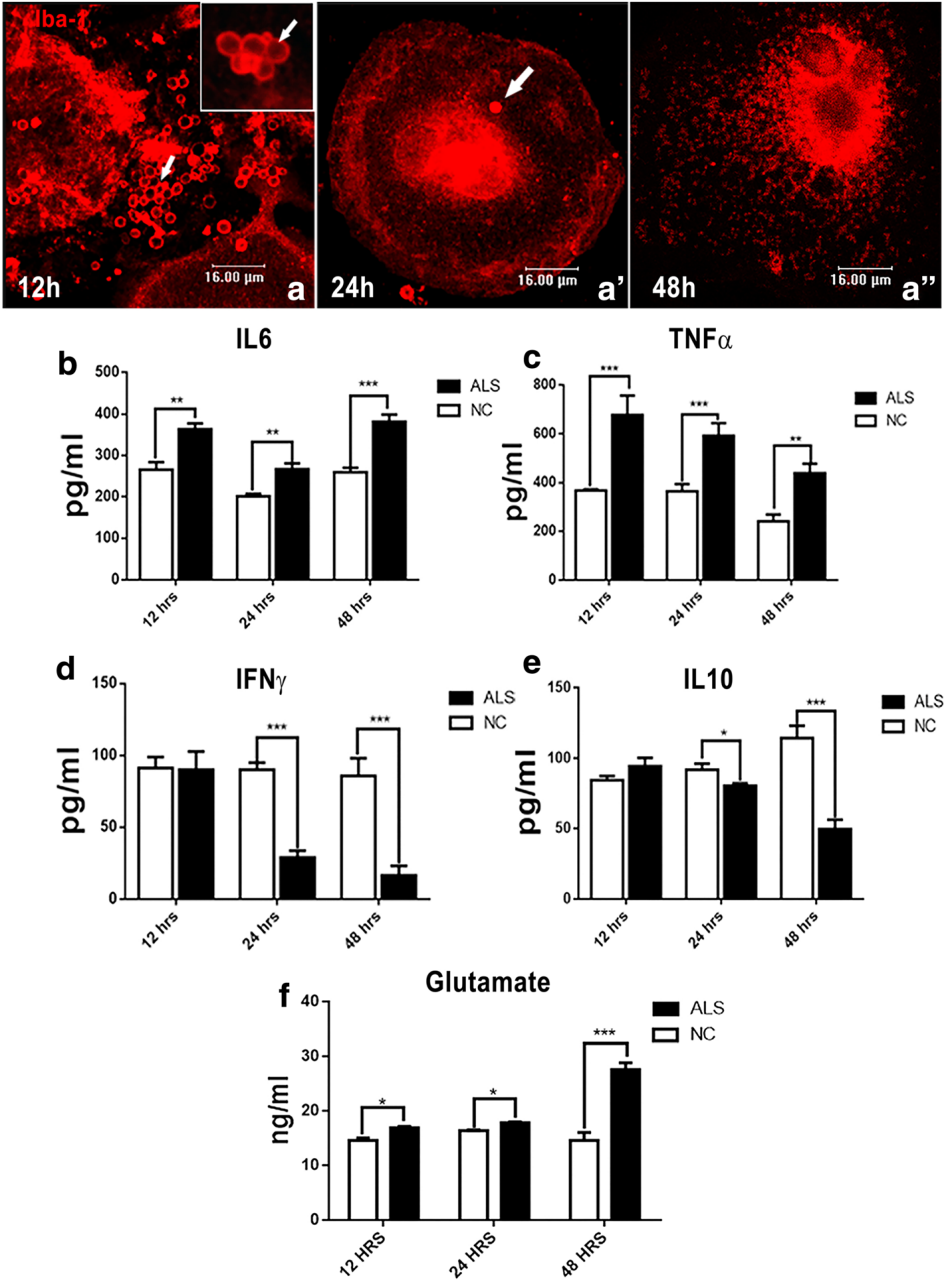
Microglial activation is temporally regulated. **a**–**a”** The Iba-1-positive microglial cells in response to ALS-CSF at 12, 24, and 48 h, respectively. Note the presence of numerous microvesicles ranging from few nanometers (arrowheads) to few micrometers (arrows) in the cultures exposed for 12 h, as compared to 24 and 48 h. The inset image shows the microvesicles at a higher magnification. A temporal analysis revealed that the pro-inflammatory cytokines including IL-6 [**b** **p < 0.01, NC vs. ALS (12 and 24 h); ***p < 0.001, NC vs. ALS (48 h); *n* = 3 in duplicates] and TNF-α [**c** ***p < 0.001, NC vs. ALS (12 and 24 h); **p < 0.01, NC vs. ALS (48 h); *n* = 3 in duplicates] were upregulated in cultures exposed to ALS-CSF for 12, 24, and 48 h, as compared to their corresponding NC. IFN-γ [**d** ***p < 0.001, NC vs. ALS (24 and 48 h); *n* = 3 in duplicates] and IL-10 [**e** *p < 0.05, NC vs. ALS (24 h); ***p < 0.001, NC vs. ALS (48 h); *n* = 3 in duplicates] levels were downregulated. **f** A surge in glutamate expression in response to ALS-CSF, starting from 12 h [*p < 0.05, NC vs. ALS (12 and 24 h); ***p < 0.001, NC vs. ALS (48 h); *n* = 3 in duplicates]. Analysis of significance was carried out using Student’s *t* test

The activation of microglia in response to ALS-CSF could be seen as early as 12 h (Fig. 2). Further, the activated microglia were predominantly surrounded by Iba-1-immunopositive vesicular structures ranging from a few nanometers to a few micrometers (Fig. 2, arrowheads and arrows, respectively) suggestive of a process akin to microvesicular shedding/release, which showed a subsequent decline at 24 and 48 h. A 24-h exposure to ALS-CSF resulted mostly in amoeboid morphology, while in addition, at 48 h, the cells also adopted a multinucleated morphology or they underwent cytorrhexis (Fig. 1e–f”). To test the functional relevance of microvesicle (MV)-like structures, we performed an analysis of the secretory profiles of microglia exposed to ALS-CSF for 12, 24, and 48 h, respectively. We observed an upregulated release of the pro-inflammatory cytokines, IL-6 and TNF-α, in the media supernatants from the ALS group, as compared to the controls as early as 12 h following exposure (Fig. 2b, c). The difference between the control and experimental groups was maximal at 12 h for TNF-α, which tapered gradually at subsequent time points. The overexpression of glutamate was more prominent following longer exposure (Fig. 2f). Concomitantly, a temporal downregulation was observed in the secretory levels of the anti-inflammatory cytokines IL-10 as well as IFN-γ (Fig. 2d, e). Out of all the time periods studied, a 48-h exposure resulted in a prolonged and sustained microglial activation and exhibited maximum morphological heterogeneity. Hence, a 48-h exposure period was chosen for further studies.

### Microglial cells respond to ALS-CSF by adopting a phenotype skewed towards pro-inflammatory pathways along with downregulated trophic support

**Fig. 3:**
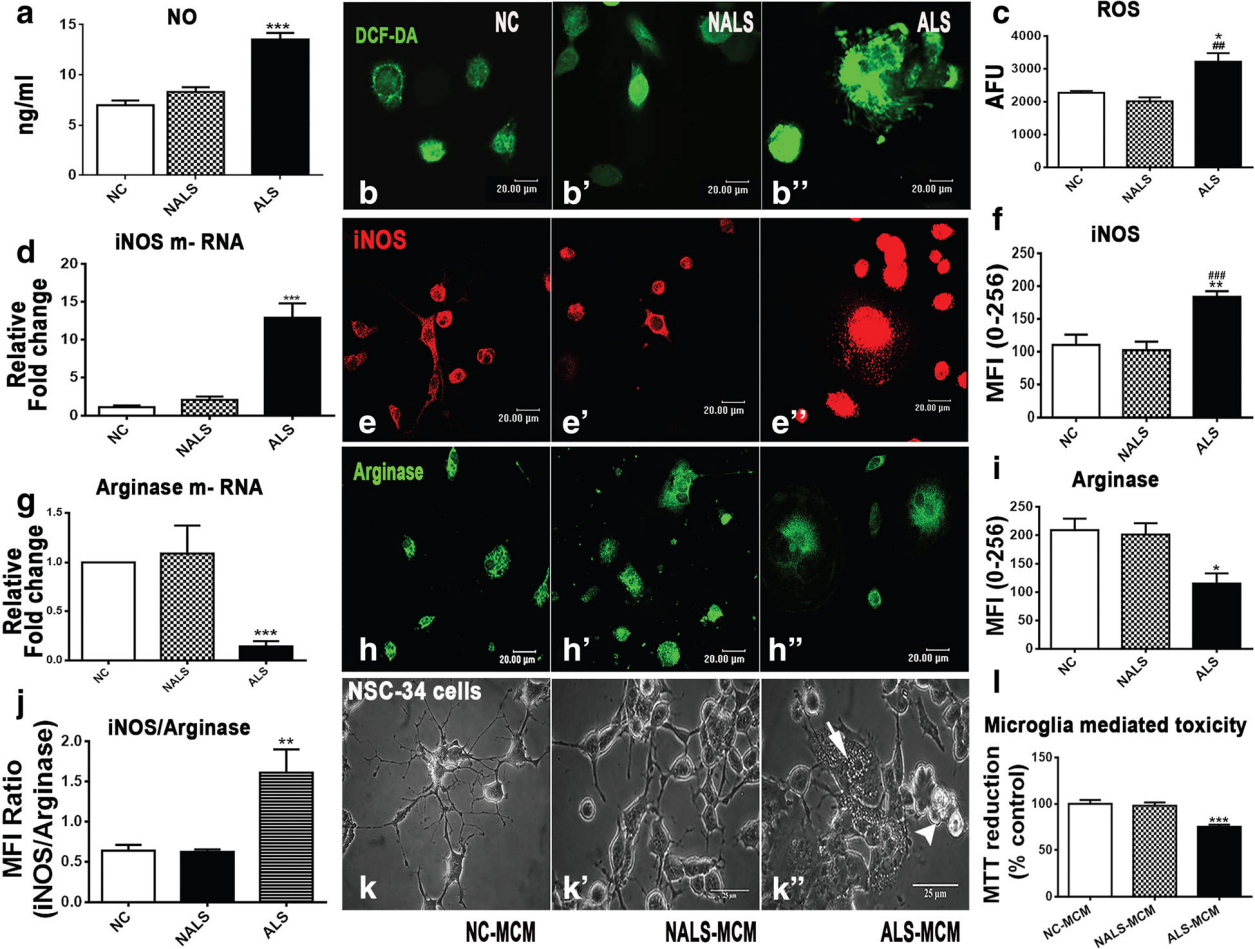
Microglial activation was predominantly neurotoxic. Microglial NO (**a** ***p < 0.001; *n* = 5 in duplicates) and ROS (**b**–**b”**, **c** *p < 0.05, NC vs. ALS; ^##^ *p* < 0.01, NALS vs. ALS; *n* = 5 in triplicates) levels were upregulated on exposure to ALS-CSF when compared to NC. iNOS mRNA (**d** ***p < 0.001; *n* = 5 in triplicates) and protein (**e**–**e”**, **f** **p < 0.01, NC vs. ALS; ^###^ *p* < 0.001, NALS vs. ALS; *n* = 5 in duplicates) expression was upregulated in a similar manner. However, arginase mRNA (**g** ***p < 0.001; *n* = 3 in triplicates) and protein (**h**–**h”**, **i** *p < 0.05; *n* = 5 in duplicates) expression was downregulated in the ALS group as compared to NC. A comparison between the iNOS and arginase protein expression clearly demonstrated a ratio skewed towards more pro-inflammatory microglial cells in the ALS group as compared to the controls (**j** **p < 0.01; *n* = 5 in duplicates). Compare the morphology of the NSC-34 cells grown in the conditioned media from the microglial cultures exposed to the control and ALS-CSF groups (ALS-MCM). Note the excessive vacuolation (**k’** white arrowhead) and clumping (**k”** white arrow) in response to the ALS-MCM. MTT reduction assay clearly showed reduced cell viability in response to the ALS-MCM (**l** ***p < 0.001; *n* = 5 in triplicates). Statistical analysis was carried out using one-way ANOVA followed by Tukey’s post hoc test

**Fig. 4:**
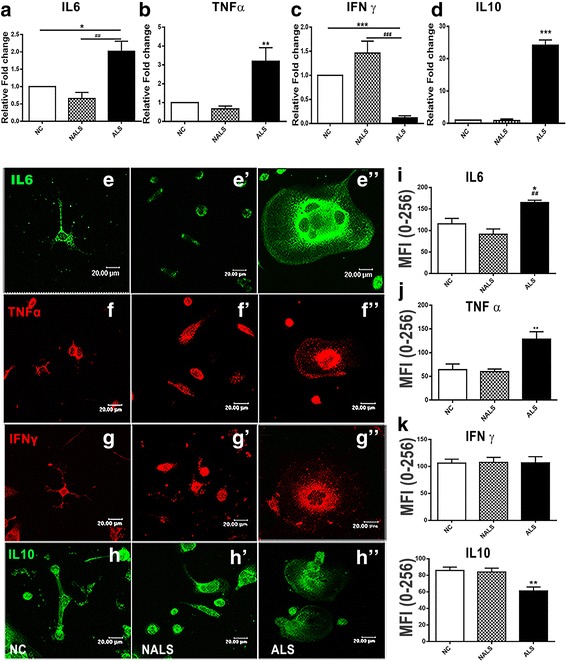
ALS-CSF regulates the synthesis and release of the cytokines. The analysis of mRNA expression uponALS-CSF exposure revealed an upregulation of IL-6 (**a** *p < 0.05, NC; ##*p* < 0.01, NALS vs. ALS; *n* = 5 in triplicates), TNF-α (**b** **p < 0.01, NC and NALS vs. ALS; *n* = 5 in triplicates), and IL-10 (**d** ***p < 0.001, NC and NALS vs. ALS; *n* = 5 in triplicates) mRNA. However, the mRNA expression of IFN-γ was downregulated (**c** ***p < 0.001, NC and NALS vs. ALS; *n* = 5 in triplicates). This figure also shows representative confocal images of microglial cultures immunolabeled for IL-6 (**e**–**e**”), TNF-α (**f**–**f”**), IFN-γ (**g**–**g”**), and IL-10 (**h**–**h”**) in the control (NC), NALS, and ALS-CSF groups. Note the prominent perinuclear localization of cytokines and multinucleation in the microglia of the ALS group. The histogram represents the mean fluorescence intensity of cytokines in all the study groups. Note the increased levels of IL-6 (**i** *p < 0.05, NC; ##*p* < 0.01, NALS vs. ALS; *n* = 5 in duplicates) and TNF-α (**j** **p < 0.01, NC and NALS vs. ALS; *n* = 5 in duplicates) in the ALS group compared to the control groups. IL-10 was downregulated (**l** **p < 0.01, NC and NALS vs. ALS; *n* = 5 in duplicates), while IFN-γ levels (**k**) remain unchanged. Statistical significance was assessed using oneway ANOVA followed by Tukey’s post hoc test

Molecular analysis of microglial activation revealed an upregulation of ROS and NO levels in cells exposed to ALS-CSF as compared to the normal controls (Fig. 3a–c). Further, the mRNA and protein expression of inducible nitric oxide synthase (iNOS), which is an inducer of pathological NO and a marker of classical activation pathway, was significantly upregulated in cultures exposed to ALS-CSF (Fig. 3d–f). The mRNA and protein expression for arginase, a marker of alternative activation, was downregulated (Fig. 3g–i). Increased iNOS/arginase ratio in response to ALS-CSF exposure for 48 h indicated that the microglial activation was skewed towards the classical pathway (Fig. 3j). Further, the conditioned medium from the microglial cells exposed to ALS-CSF induced cell death in NSC-34 cells, suggesting the existence of neurotoxic potential in activated microglial cells as a result of exposure to ALS-CSF (Fig. 3k–l). We studied the mRNA expression as well as cellular localization of the inflammatory markers after 48 h of ALS-CSF exposure. An upregulation was registered in the mRNA as well as the protein levels of IL-6 (Fig. 4a, e–e”, i) and TNF-α (Fig. 4b, f–f”, j), while IFN-γ mRNA was downregulated with no significant changes observed in its cellular protein expression (Fig. 4c, g–g”, k) in the ALS group as compared to the normal and disease controls. While a significant upregulation was seen in the IL-10 mRNA levels in response to ALS-CSF, the cellular protein expression showed a downregulation, which could explain the decreased secretion of IL-10 in cultures exposed to ALS-CSF (Fig. 4d, h–h”, l).

**Fig. 5:**
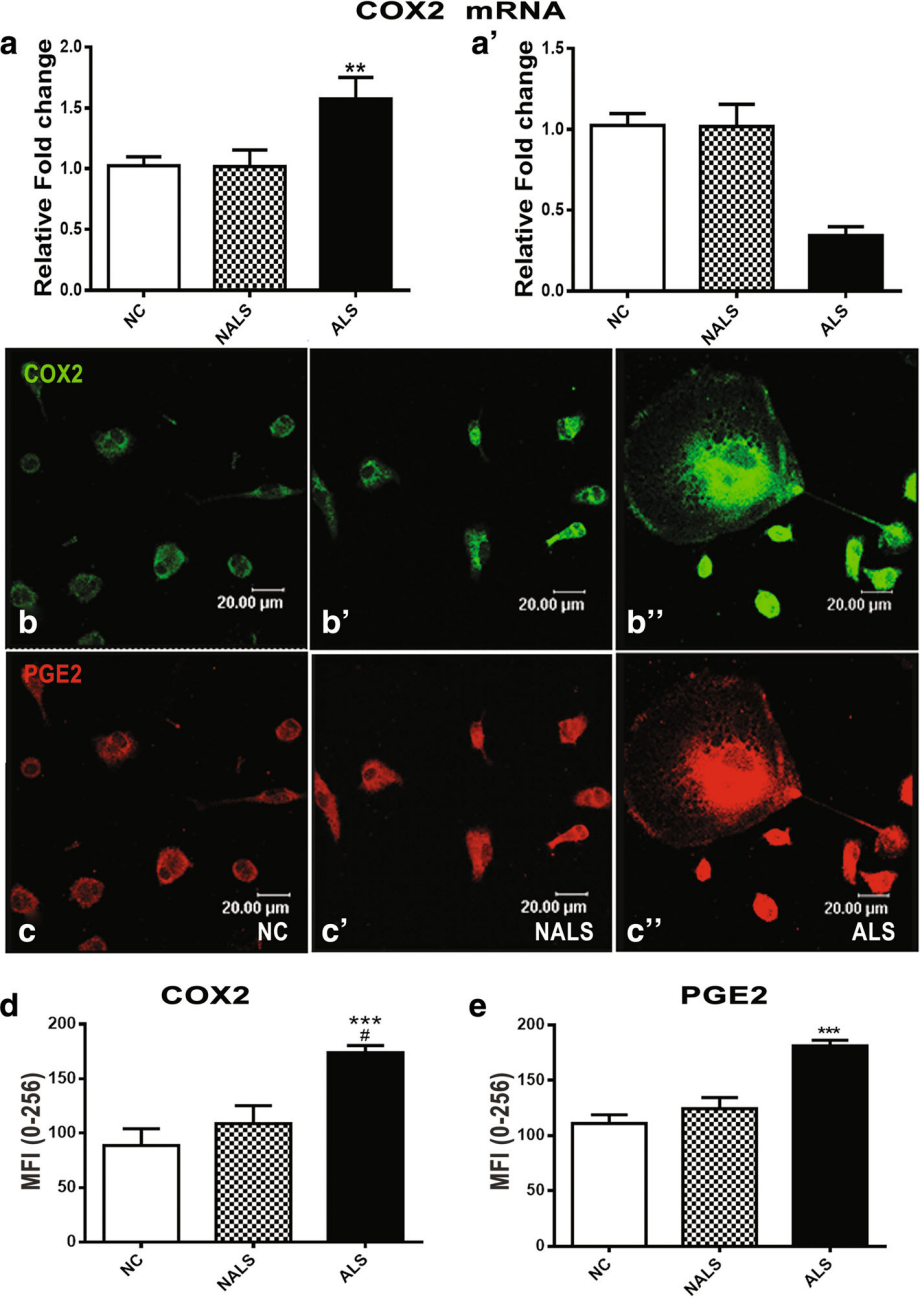
Microglia reacts to ALS-CSF by acquiring an inflammatory phenotype. Analysis of COX-2 mRNA levels after 48 h of exposure to ALS-CSF revealed a significant upregulation in three samples (**a** **p < 0.01, NC and NALS vs. ALS) and downregulation in the other two (**a’**) in response to ALS-CSF. Representative confocal images of cultures immunostained for COX-2 (FITC, green; **b**–**d** ***p < 0.001, NC; ^##^ *p* < 0.01, NALS vs. ALS; *n* = 5 in duplicates; graph **e**) and its downstream inflammatory molecule PGE2 (Cy3, red, **b**–**d”** ***p < 0.001, NC and NALS; *n* = 5 in duplicates; graph **f**). Analysis of the significance was carried out using one-way ANOVA followed by Tukey’s post hoc test

ALS-CSF also induced changes in inflammatory markers such as COX-2 and PGE2. With regard to mRNA expression of COX-2, three out of five ALS-CSF samples studied showed a significant upregulation of COX-2 mRNA, while two showed downregulation compared to the controls (Fig. 5a, a’). However, all the five ALS-CSF samples showed a significant upregulation of the cellular expression of COX-2 as well as PGE2, when compared to the normal and disease controls (Fig. 5b–e).

**Fig. 6:**
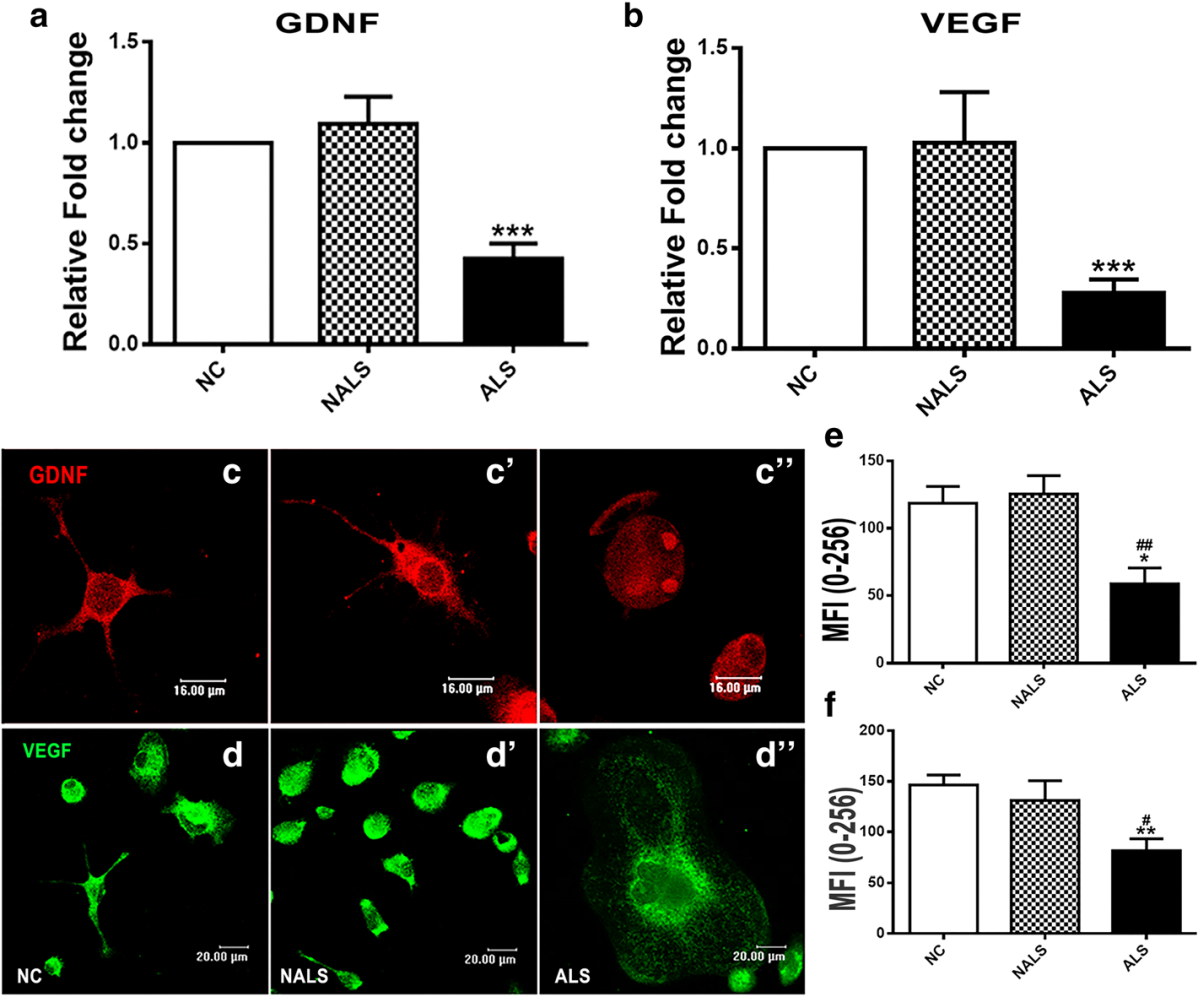
Downregulation of trophic factors in ALS-CSF-exposed microglial cultures. Analysis of mRNA expression showed a downregulation of both GDNF (**a** ***p < 0.001, NC and NALS vs. ALS; *n* = 3 in triplicates) and VEGF (**b** ***p < 0.001, NC and NALS vs. ALS; *n* = 3 in triplicates) upon exposure to ALS-CSF. A quantitative analysis of the MFI of immunolabeled proteins further corroborated the findings of overall downregulation of GDNF (**c**–**c”**, graph **e**, *p < 0.05, NC; ^##^ *p* < 0.01, NALS vs. ALS; *n* = 5 in duplicates) and VEGF (**d**–**d”**, graph **f**, **p < 0.01, NC; ^#^ *p* < 0.05, NALS vs. ALS; *n* = 5 in duplicates) expression in response to ALS-CSF. Statistical significance was calculated using one-way ANOVA followed by Tukey’s post hoc test

Further, ALS-CSF exposure also resulted in a significant downregulation of the mRNA as well as protein expression of the trophic factors, namely GDNF (Fig. 6a, c–c”, e) and VEGF (Fig. 6b, d–d”, f, f”), when compared to the normal and disease controls. This indicated a reduced trophic support provided by the microglial cells along with the neuroinflammatory response to the toxic factors present in the ALS-CSF.

### Chitotrisidase-1, the factor prominently upregulated in ALS-CSF, acts selectively on microglial cells

**Fig. 7:**
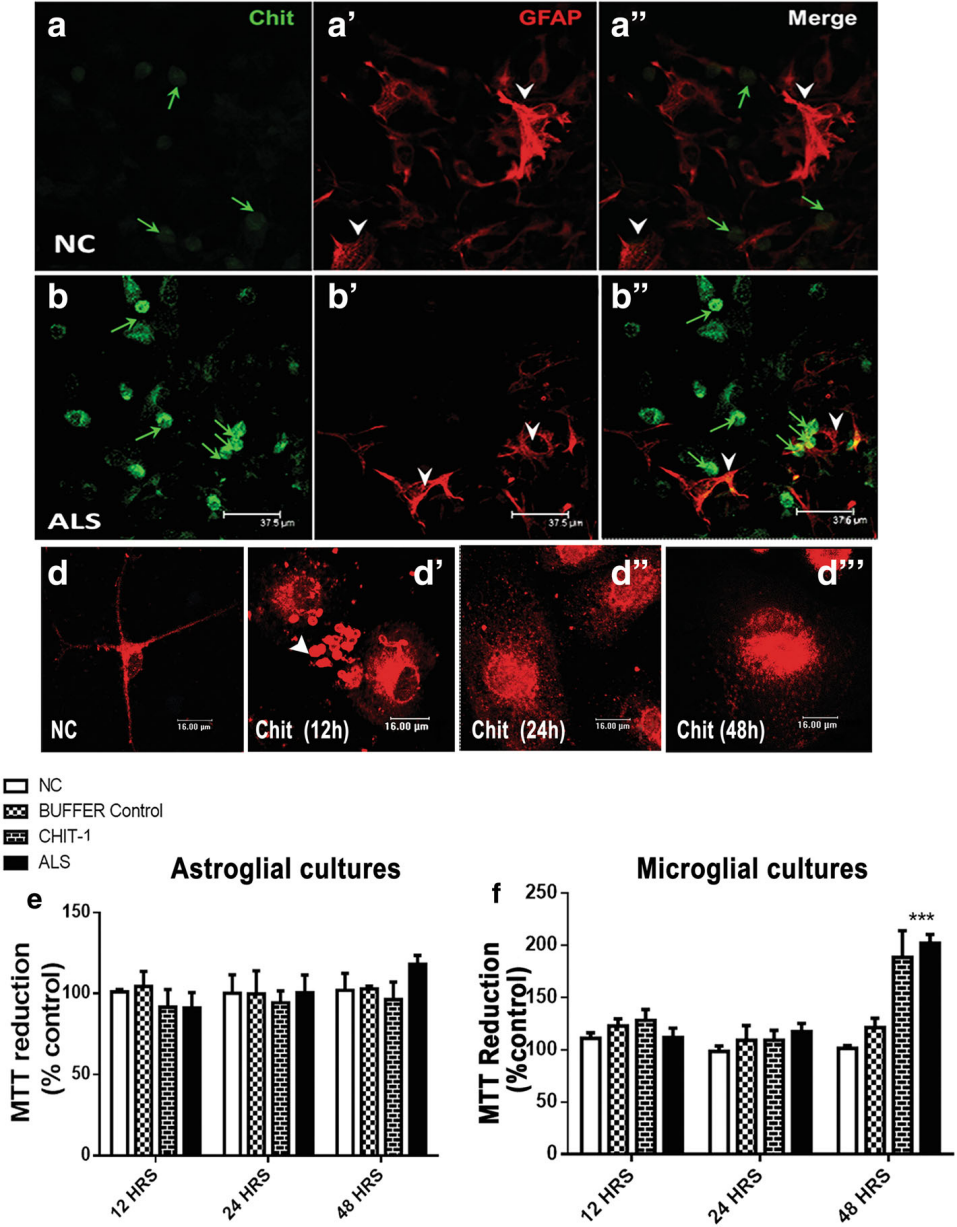
CHIT-1, the factor upregulated in ALS-CSF, acts upon and is mediated via microglia. Representative confocal images of the mixed glial cultures (**a**–**b”**) immunolabeled for CHIT-1 (green) and GFAP (red) in control and ALS-CSF-exposed cultures. Note the absence of CHIT-1 immunoreactivity in the GFAP-positive astrocytes (**a’**, **a”**, **b’**, **b”** white arrowheads), while a subset of non-GFAP cells expressed CHIT-1 in ALS-CSF-exposed cultures (**b**, **b”**). **d**–**d”** Representative phase contrast and confocal images of the microglial cultures immunolabeled for Iba-1 in normal control (**d**) and the subsets exposed to CHIT-1 for 12, 24, and 48 h, respectively (**d’**–**d”’**). Note the increased number of microglia and their transformation from resting to amoeboid stages. Also, note the presence of microvesicle-like structures (arrowhead) in cultures exposed for 12 h, similar to that in ALS-CSF-exposed cultures. Histograms **e** and **f** depict the effect of exposure to CHIT-1 on MTT reduction by astroglial and microglial cultures, respectively. Note the significant increase in MTT reduction by the microglial cells exposed to CHIT-1 for 48 h (**f** ***p < 0.001, NC and buffer control vs. CHIT-1; *n* = 5 in triplicates). The CHIT-1 exposure did not have a significant effect on the viability of astrocytes

We previously reported a multifold increase in the concentration of CHIT-1 in ALS-CSF and its enhanced expression in microglia in response to ALS-CSF [33]. We also reported the induction of inflammation in astroglia following exposure to ALS-CSF [36]. To further delineate whether the CHIT-1 expression is specific to microglia or also present in activated astrocytes, we studied CHIT-1 expression in the mixed glial cultures exposed to ALS-CSF. While CHIT-1 immunoreactivity was clearly absent in the GFAP-positive astrocytes in both normal controls and ALS group, the non-GFAP-positive cells showed enhanced CHIT-1 expression upon ALS-CSF exposure, thus corroborating our previous findings of microglial cells as the primary source of CHIT-1 in CSF (Fig. 7a–b”).

Further, in order to investigate the direct effects of CHIT-1, we exposed the microglial cells to the concentrations of CHIT-1 similar to that found in ALS-CSF. Exposure to CHIT-1 caused a microglial transformation similar to that seen in the ALS group, with the presence of MV-like structures prominent at 12 h, while other transformations including MGCs and cytorrhexis were found at 48 h (Fig. 7c–c”’). An increase in the MTT reduction was observed in the enriched microglial cultures exposed to CHIT-1, in a manner similar to ALS-CSF, thereby indicating a direct action of CHIT-1 on microglial activation (Fig. 7e). The apparent specificity of CHIT-1 action on microglia was further supported by the absence of any significant effect on the viability of primary astrocytes (Fig. 7e).

## Discussion

Microglial activation is a dynamic process and may involve various temporally, physiologically, and/or spatially regulated events that govern the morphological and functional changes observed in the reactive microglia [6, 42, 43]. The pro-inflammatory microglial transformation observed in response to ALS-CSF indicates an inflammatory microenvironment of the CNS in SALS that may be further dynamically regulated by the actively transforming microglia, in a closed, amplifying feedback loop. The ability of the conditioned media from the microglial cultures exposed to ALS-CSF to exert toxicity to the NSC-34 cells corroborates the non-cell autonomous disease propagation in ALS [42]. Recent studies discuss the importance of MV/exosomes in the neuronal spread of TDP-43 proteinopathies in ALS [44, 45]. Studies conducted on the glial MVs released by the reactive astrocytes and glioma cells hint towards a possible engagement of MVs in the intercellular communication [46, 47]. Further, investigations on microglial microvesicular release in neuroinflammation also suggest its participation in disseminating neurotoxicity via proteins and/or microRNAs [48, 49]. The reports of pro-inflammatory nature of microglial MVs, especially at early time points (8 h) as opposed to the anti-inflammatory nature at later stages (> 24 h), support our observations of early pro-inflammatory secretion and concomitantly increased vesicular structures in the ALS-CSF-exposed microglial cultures [50, 51]. Interestingly, at 48 h, ALS-CSF also induced multinucleation and cytorrhexis in the microglial cultures, which hints at dysregulated pathways leading to failed cytokinesis and overactivation-induced self-moderation [52, 53]. Such a phenomenon has also been reported in another study involving the mutant SOD1 mice model, where it is explained as an accidental product of the *high-intensity neuroinflammation* [41]. Induction of NF-κB, the inflammatory regulator for glial activation, has been documented in the autopsy studies as well as TDP-43 models [54–56]. Further, studies involving the C9orf72 pathology suggest towards a potential role of the microglial C9orf72 repeat expansions in exacerbating neuroinflammation, resulting in a loss-of-function endosomal-lysosomal aberration, thus favoring a pro-inflammatory microglial phenotype [57]. These observations highlight the role of neuroinflammation in the disease pathology irrespective of the nature of the etiogenesis.

Upregulation of microglial inflammatory mediators and retraction of the beneficial trophic support demonstrate the ability of ALS-CSF to shift the microglial functionality predominantly towards an activated pro-inflammatory physiology that is able to propagate inflammation by sending inflammatory signals in the circulation. Overexpression of iNOS, along with the downregulation of arginase, may suggest the induction of a pathological switch towards physiologically inflamed or the classically activated (M1) microglia in response to ALS-CSF [58]. This correlated with the increased expression and secretion of IL-6 and TNF-α, as well as a temporal decline in the secretion of the neuroprotective cytokines IL-10 and IFN-γ. However, the upregulation of IL-10 mRNA levels and the differential regulation of COX-2 mRNA in response to different ALS-CSF samples, contrary to their pro-inflammatory protein expression patterns, also depict a *conflicting state* of activation that may defy the typical M1/M2 polarization. Studies conducted in the mutant SOD1 mice model also suggested a disease-specific microglial profile, where a simultaneous dysregulation was reported in the expression of M1- and M2-specific markers in early stages, with either a slight bias [10] towards the M1 type or none [17] at the later stages. Since in our study the overall findings support the neurotoxic outcomes, we propose that these profiles may arise either as the result of a dynamic, reversible switch between a pro- and anti-inflammatory phenotype or a failed attempt of the repair processes at work.

l-Glutamate release from activated microglia downregulates astroglial glutamate transporter expression [59], a pathological phenomenon well documented in ALS [60]. Apart from this, the elevated microglial glutamate may also exacerbate neurodegeneration through dysregulated gliotransmission via purinergic receptors and Ca^2+^ wave propagation [61] or induce necrosis and necroptosis as seen in response to ALS-CSF [27, 38]. Alternatively, excess glutamate can also accentuate neuroinflammation by interacting with inflammatory modulators including ROS [62], PGE2 [63], and NO [64], which were found elevated in response to the ALS-CSF. The neurotoxic potential of the conditioned media from microglial cultures exposed to the ALS-CSF (ALS-MCM) corroborated the pro-inflammatory role of the factors predominantly upregulated in ALS-MCM, including IL-6, TNF-α, and NO. Further, the higher quantum of TNF-α levels, specifically in the initial phase, suggests its role as a potent, early initiator of neuroinflammation, much in accordance with the studies conducted in mSOD1 models [65, 66]. Recent literature elucidates the neuroprotective nature of microglial IFN-γ signaling in CNS pathology through the recruitment of immunoregulatory cells including macrophages, and their dysregulation in the neurodegenerative disorders [4, 67, 68]. The downregulation of microglial IFN-γ secretion may thus suggest a reduced anti-inflammatory leucocyte infiltration leading to the aggravation of the disease pathology.

Apart from a reduced trophic support, downregulation of microglial VEGF has been shown to correspond with a phagocytic microglial phenotype [69]. In a similar manner, a study by Matsushita and colleagues also reported a significant downregulation of GDNF in response to endotoxin LPS, suggesting that the reduction in GDNF expression could be a feature of inflammation [70]. These studies highlight the protective roles of both these trophic factors in disease pathogenesis through inhibition of inflammation and provide sufficient evidence to consider their relevance in clinical applications.

These observations clearly suggest that following exposure to ALS-CSF, microglial cells are activated and adopt a toxic phenotype. Additionally, we have provided compelling evidence inflammatory response of the spinal cord-derived microglia to ALS-CSF-mediated insult. Interestingly, in contrast to the early microglial activation, the maximal degenerative changes in the neurons exposed to ALS-CSF were observed at 48 h [27]. Similarly, the first known astroglial response to ALS-CSF in terms of gliosis or cytokine secretion was not observed before 24 h and became prominent at 48 h. The present observations, along with the previously reported, marginally delayed, inflammatory behavior of astrocytes towards ALS-CSF, might suggest a synergistic interplay between microglia and astrocytes in potentiating, sustaining, and aggravating the insult [36].

In addition, the present study attempts to explain the continued failure of anti-inflammatory therapeutic approaches in the clinical trials, despite obvious pathological contribution of inflammation in ALS [71, 72]. Since the alterations observed in the microglial physiology can at best be described as multifactorial with the interplay of components like pro-inflammatory and anti-inflammatory cytokines, PGE2, ROS, and glutamate, mitigating inflammation by blocking specific molecules/pathways would be inefficient. Moreover, the lacuna with employing anti-inflammatory drugs to target inflammation in general lies in the controversial observations of varied effects of these drugs, ranging from amelioration to exacerbation of the disease pathology, in different conditions, thus rendering them unsuitable for therapeutic interventions. For instance, minocycline was reported to be protective in the animal studies during the initial stages, as well as in the clinical trials at specific dosage, and stages of the disease, but had detrimental effects in the later stages or in higher doses [72–74]. Hence, it is imperative to thoroughly investigate the mechanism of microglial inflammation in order to pin down the exact pathways affected in ALS, and design a combinatorial approach to target microglial activation and neuroinflammation.

CHIT-1, the protein primarily upregulated in the ALS-CSF, is considered as a marker of chronic activation of macrophages in the peripheral system [75]. Although CHIT-1 has also been implicated in various neurological diseases, its function in the CNS pathology remains least understood. While CHIT-1 is shown to have a pro-inflammatory potential in neurological disorders like stroke [76], its neuroprotective role in the inflammatory conditions like multiple sclerosis has also been suggested [77]. The microglial cell-specific upregulation of CHIT-1 expression in response to ALS-CSF suggests the possibility of neuroinflammatory process in ALS patients, led by the chronically activated microglia. The selective action of CHIT-1 on microglial cells suggests a vicious cycle of neurodegeneration primarily mediated by microglial cells and propagated to others via a cell-to-cell communication and/or circulating fluids. Since the reactive microglia in ALS are derived from the endogenous pool and not from the circulating monocytes [10], the endogenous microglial population may act as the predominant source of CHIT-1 in ALS-CSF, thus rendering them as potential clinical targets.

## Conclusion

**Fig. 8:**
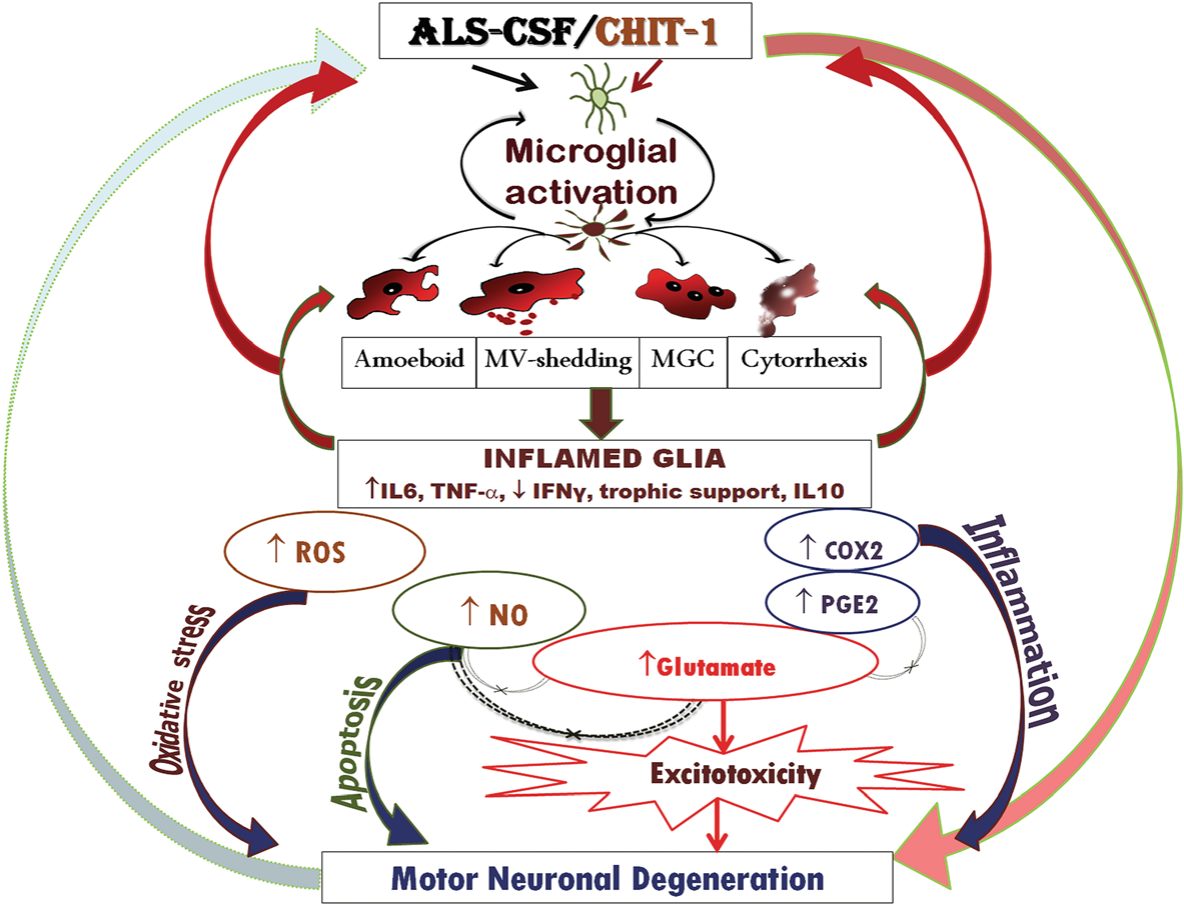
Schematic representation of microglial involvement in the ALS-CSF/CHIT-1-induced neurotoxicity. Microglial activation marks the early event of SALS pathology in response to the inflammatory trigger through ALS-CSF/CHIT-1 exposure. It results in the vicious cycle leading to activation/overactivation of microglial cells, followed by morphological and functional transformation of the microglial cells to encourage neuroinflammation. The activation leads to a *push-forward* mechanism, encouraging the overproduction of pro-inflammatory cytokines as an early event, subsequently leading to the production of PGE2, ROS, NO, and glutamate, while downregulating beneficial trophic factors and anti-inflammatory factors. These events may lead to motor neuron degeneration through interlinked pathways. *MGC* multinucleated giant cell MV shedding = activated microglia engaged in shedding microvesicle-like structures; cytorrhexis = cytoplasmic fragmentation resulting from microglial death due to overactivation

Based on our findings, we propose that microglial pathology in SALS is morphologically and functionally dynamic, leading to the neurodegeneration through myriad pathways (Fig. 8). It appears to operate by a *push-forward* mechanism, where microglia are the first to strike, possibly accompanied by astrocytes, thus setting up a neuroinflammatory cascade and eventually leading to accentuated neurodegeneration. In the light of the above, a combinatorial therapeutic approach is warranted, by suppressing the pro-inflammatory pathways and promoting the anti-inflammatory role of microglia.

## Additional file

Additional file 1: Details of the CSF samples taken for the study. Table S1 describes the age and gender of the subjects, duration of disease, nature of disease progression, symptoms, onset pattern and chit-1 levels. Table S2 provides details about the age and gender matched NALS-CSF samples investigated in the present study. (DOC 41 kb)

## Abbreviations

ALS: Amyotrophic lateral sclerosis
ALS-CSF: Cerebrospinal fluid of amyotrophic lateral sclerosis patients
ALS-CSF-MCM: Amyotrophic lateral sclerosis cerebrospinal fluid-exposed microglial cell-conditioned medium
ANOVA: Analysis of variance
CHIT-1: Chitotriosidase-1
CHIT-1-MCM: Chitotriosidase-1-exposed microglial cell-conditioned medium
CNS: Central nervous system
COX-2: Cyclooxygenase-2
DCFDA: Dichlorofluorescin diacetate
FALS: Familial amyotrophic lateral sclerosis
GDNF: Glial cell line-derived neurotrophic factor
GFAP: Glial fibrillary acidic protein
IFN-γ: Interferon-γ
IL-6: Interleukin-6
IL-10: Interleukin-10
iNOS: Inducible nitric oxide synthase
MGC: Multinucleated giant cell
mRNA: Messenger RNA
MS: Multiple sclerosis
MTT: 3,4,5-Dimethylthiazol-2-yl)-2,5-diphenyltetrazolium bromide
MV: Microvesicle
NALS-CSF: CSF of non-ALS patients
NC: Normal control
NO: Nitric oxide
PGE2: Prostaglandin E2
ROS: Reactive oxygen species
SALS: Sporadic amyotrophic lateral sclerosis
SOD1: Superoxide dismutase 1
TAR: Transactive response
TDP-43: 43-kDa transactive response DNA-binding protein
TNF-α: Tumor necrosis factor alpha
VEGF: Vascular endothelial growth factor
