# Supplementary material for: Etiogenic factors present in the cerebrospinal fluid from amyotrophic lateral sclerosis patients induce predominantly pro-inflammatory responses in microglia
Source: J Neuroinflammation. 2017 Dec 16;14:251. doi: 10.1186/s12974-017-1028-x (PMC5732516; doi:10.1186/s12974-017-1028-x)
Supplement: Additional file 1: — Details of the CSF samples taken for the study. Table S1 describes the age and gender of the subjects, duration of disease, nature of disease progression, symptoms, onset pattern and chit-1 levels. Table S2 provides details about the age and gender matched NALS-CSF samples investigated in the present study. (DOC 41 kb) [file 12974_2017_1028_MOESM1_ESM.doc]

**Additional File 1.**

**Table 1: Details of ALS-CSF:**

| **Gender** | ***Females***: 2 (40%)  ***Males***: 3 (60%) |
| --- | --- |
| **Age at presentation (Mean ± SD)** | 50± 9.95 (41 – 65) Years |
| **Age at onset (Mean ± SD)** | 48.96± 9.80 (40-64) Years |
| **Duration of illness (Mean ± SD)** | 12.4 ± 6.80 (7.0 – 24) Months |
| **Onset Pattern:** | ***Limb:*** 2 (40%)  ***Bulbar***: 3(60%) |
| **Symptoms** | ***Lower limb involvement***: 2(40%)  ***Upper limb involvement***: 3(60%)  ***Dysarthria:***2 (40%)  ***Dysphagia:*** 3 (60%)  ***Spasticity:*** 3 (60%) with evidence of pyramidal signs in the form of spasticity and exaggerated Deep Tendon Reflexes |
| **Disease Severity** | ***Mild***: 2 (40 %)  ***Moderate***: 3 (60%) |
| **Chitotrisidase levels (Mean ± SD)** | 8123.67±3308.54 pg/ml (published previously [1]) |

**Table 2**: Details of NALS-CSF:

| **Gender** | ***Females***: 2 (40%)  ***Males***: 3 (60%) |
| --- | --- |
| **Age at presentation (Mean ± SD)** | 50.4 ± 2.88 (47 – 55) Years |
| **Diagnosis** | Intracranial hypertension 2 (40%)  Normal pressure hydrocephalus: 2 (40%)  Peripheral neuropathy 1(20%) |

1. Varghese AM, Sharma A, Mishra P, Vijayalakshmi K, Harsha HC, Sathyaprabha TN, Bharath SM, Nalini A, Alladi PA, Raju TR**: Chitotriosidase - a putative biomarker for sporadic amyotrophic lateral sclerosi**s*. Clin Proteomic*s 2013**, 1**0:19.
